# Bile acids potentiate proton‐activated currents in *Xenopus laevis* oocytes expressing human acid‐sensing ion channel (ASIC1a)

**DOI:** 10.14814/phy2.13132

**Published:** 2017-02-13

**Authors:** Alexandr V. Ilyaskin, Alexei Diakov, Christoph Korbmacher, Silke Haerteis

**Affiliations:** ^1^ Institut für Zelluläre und Molekulare PhysiologieFriedrich‐Alexander‐Universität Erlangen‐Nürnberg (FAU)ErlangenGermany

**Keywords:** Acid‐sensing ion channel 1a (ASIC1a), bile acids, degenerin site, patch clamp

## Abstract

Acid‐sensing ion channels (ASICs) are nonvoltage‐gated sodium channels transiently activated by extracellular protons and belong to the epithelial sodium channel (ENaC)/Degenerin (DEG) family of ion channels. Bile acids have been shown to activate two members of this family, the bile acid‐sensitive ion channel (BASIC) and ENaC. To investigate whether bile acids also modulate ASIC function, human ASIC1a was heterologously expressed in *Xenopus laevis* oocytes. Exposing oocytes to tauro‐conjugated cholic (t‐CA), deoxycholic (t‐DCA), and chenodeoxycholic (t‐CDCA) acid at pH 7.4 did not activate ASIC1a‐mediated whole‐cell currents. However, in ASIC1a expressing oocytes the whole‐cell currents elicited by pH 5.5 were significantly increased in the presence of these bile acids. Single‐channel recordings in outside‐out patches confirmed that t‐DCA enhanced the stimulatory effect of pH 5.5 on ASIC1a channel activity. Interestingly, t‐DCA reduced single‐channel current amplitude by ~15% which suggests an interaction of t‐DCA with a region close to the channel pore. Molecular docking predicted binding of bile acids to the pore region near the degenerin site (G433) in the open conformation of the channel. Site‐directed mutagenesis demonstrated that the amino acid residue G433 is critically involved in the potentiating effect of bile acids on ASIC1a activation by protons.

## Introduction

The epithelial sodium channel (ENaC)/degenerin (DEG) superfamily of ion channels encompasses more than 60 members including the acid‐sensing ion channels (ASICs) (Kellenberger and Schild 2015). ASICs are nonvoltage‐gated sodium channels transiently activated by a rapid increase in the extracellular concentration of protons (acidic pH) (Waldmann et al. 1997; Carattino 2011; Kellenberger and Schild 2015). Proposed functions of ASICs include peripheral perception of pain, taste, and mechanosensation (Deval et al. 2010; Wemmie et al. 2013). So far, 8 human ASIC subunits have been cloned (termed ASIC1a, ‐1b; ASIC2a, ‐2b; ASIC3a, ‐3b, ‐3c; ASIC4) which are encoded by four ASIC genes (Kellenberger and Schild 2015). All ASIC subunits share a common membrane topology which is characterized by a large extracellular domain, short intracellular amino and carboxy termini, and two transmembrane regions (TM1 and TM2) (Canessa 2007). It is thought that individual ASIC subunits associate as homo‐ or heteromeric complexes. Chicken ASIC1 (90% sequence identity with human ASIC1a) has been crystallized as a homotrimer (Jasti et al. 2007; Gonzales et al. 2009; Baconguis and Gouaux 2012; Dawson et al. 2012; Baconguis et al. 2014), but there is also evidence for a homotetrameric assembly state of human ASIC1a at the cell surface (van Bemmelen et al. 2015). Thus, at present the subunit stoichiometry of ASIC remains a matter of debate.

Protons seem to be the only physiological activators of ASICs. However, several modulators of ASIC function such as neuropeptides, polyamines, and arachidonic acid have been described (Smith et al. 2007; Sherwood and Askwith 2009; Duan et al. 2011; Wemmie et al. 2013). Recently, it has been shown that bile acids can activate two members of the ENaC/DEG superfamily of ion channels, namely the bile acid‐sensitive ion channel (BASIC) (Wiemuth et al. 2012; Lefevre et al. 2014) and ENaC (Wiemuth et al. 2014; Ilyaskin et al. 2016). The aim of this study was to investigate whether bile acids can also modulate the function of human ASIC1a heterologously expressed in *Xenopus laevis* oocytes.

## Materials and Methods

### Materials

Sodium tauro‐chenodeoxycholate (t‐CDCA), tauro‐deoxycholate (t‐DCA), and tauro‐cholate (t‐CA) were purchased from Sigma‐Aldrich (Taufkirchen, Germany). n‐Dodecyl‐*β*‐D‐maltoside was obtained from ThermoFisher (Rockford, IL).

### Plasmids

Full‐length cDNA for human ASIC1a was kindly provided by Professor John Michael Edwardson (Department of Pharmacology, University of Cambridge, UK). It was subcloned into the pcDNA3.1 vector. Linearized plasmids were used as templates for cRNA synthesis using T7 RNA polymerases (mMessage mMachine, Ambion, Austin, TX). The single‐point mutations (G433C and G443S) were introduced in ASIC1a using QuikChange II site‐directed mutagenesis kit (Stratagene, La Jolla, CA). Sequences were confirmed by sequence analysis (LGC Genomics, Berlin, Germany).

### Isolation of oocytes and two‐electrode voltage‐clamp experiments

Isolation of *Xenopus laevis* oocytes and two‐electrode voltage‐clamp experiments were performed essentially as described previously (Diakov and Korbmacher 2004; Haerteis et al. 2009, 2014; Diakov et al. 2010; Ilyaskin et al. 2016). Defolliculated stage V‐VI oocytes were obtained from ovarian lobes of adult female *Xenopus laevis* in accordance with the principles of German legislation, with approval by the animal welfare officer for the University of Erlangen‐Nürnberg, and under the governance of the state veterinary health inspectorate. Animals were anesthetized in 0.2% MS222 (Sigma, Taufkirchen, Germany) and ovarian lobes were obtained by a small abdominal incision. Oocytes were injected with wild‐type or G433S mutant ASIC1a cRNA using 0.5–2 ng or with G433C mutant ASIC1a using 5–10 ng per oocyte. Injected oocytes were incubated in ND96 solution (in mmol/L: 96 NaCl, 2 KCl, 1.8 CaCl_2_, 1 MgCl_2_, 5 HEPES, pH 7.4 with Tris) supplemented with 100 units/ml sodium penicillin and 100 μg/mL streptomycin sulfate. Oocytes were studied 48 h after cRNA injection. Modified ND96 solution was used as bath solution (in mmol/L: 96 NaCl, 4 KCl, 1 CaCl_2_, 1 MgCl_2_, 10 HEPES, pH 7.4 or 5.5 adjusted with Tris). Sodium salts of bile acids were directly dissolved in bath solution without significantly changing solution pH. A gravity‐fed system was used for bath solution exchanges controlled by a magnetic valve system (ALA BPS‐8) in combination with a TIB14 interface (HEKA). An individual oocyte was placed in an experimental chamber with a narrow flow channel (length: 45 mm, height: 3 mm, width: 3 mm) with a U‐shaped cross section of ~8 mm^2^. The oocyte was positioned in the channel close to the site of solution inflow and was held in place by the impaling microelectrodes. To achieve rapid and reproducible solution exchanges at the oocyte, the perfusion rate was carefully adjusted for each experimental solution to ~10 mL/min which resulted in a flow velocity of ~20 mm/sec. The flow channel drained into a reservoir (2 cm × 1 cm) from which the solution was continuously removed *via* a suction tube. The suction tube was adjusted to maintain the fluid level at ~2 mm.

### Single‐channel recordings in outside‐out patches

Single‐channel recordings in outside‐out membrane patches of ASIC1a expressing oocytes were performed 48 h after cRNA injection using conventional patch clamp technique essentially as described previously (Diakov and Korbmacher 2004; Diakov et al. 2008; Lefevre et al. 2014; Ilyaskin et al. 2016). Patch pipettes were pulled from borosilicate glass capillaries and had a tip diameter of about 1–1.5 μm after fire polishing. Pipettes were filled with K‐gluconate pipette solution (in mmol/L: 90 K‐gluconate, 5 NaCl, 2 Mg‐ATP, 2 EGTA, and 10 HEPES, pH 7.2 with Tris). Seals were routinely formed in a low sodium NMDG‐Cl bath solution (in mmol/L: 95 NMDG‐Cl, 1 NaCl, 4 KCl, 1 CaCl_2_, 1 MgCl_2_, 10 HEPES, 7.4 pH with Tris). In this bath solution, the pipette resistance averaged about 7 MΩ. After seal formation, the NMDG‐Cl solution was switched to a NaCl bath solution in which NMDG‐Cl (95 mmol/L) was replaced by NaCl (95 mmol/L). The holding potential was set to −70 mV. Using a 3 mol/L KCl flowing boundary electrode, the liquid junction (LJ) potential occurring at the pipette/NaCl bath junction was measured to be 12 mV (bath positive) (Lefevre et al. 2014). Thus, at a holding potential of −70 mV the effective transpatch potential was −82 mV. This value is close to the calculated equilibrium potential of Cl^−^ (E_CI_^−^ = −77.4 mV) and K^+^ (E_K_^+^ = −79.4 mV) under our experimental conditions. Experiments were performed at room temperature (~23°C). The excised patch at the tip of the pipette was positioned centrally in the fluid inflow to the experimental chamber. Flow rates were adjusted to allow rapid solution exchanges at the patch with a flow velocity of ~20 mm/sec. Single‐channel current data were initially filtered at 2 kHz and sampled at 6 kHz. The current traces were refiltered at 400 Hz to resolve the single‐channel current amplitude (*i*). The apparent number of active channels in a patch was determined by visual inspection of the current traces. Single‐channel data were analyzed using the program “Nest‐o‐Patch” (http://sourceforge.net/projects/nestopatch) written by Dr. V. Nesterov (Institut für Zelluläre und Molekulare Physiologie, Friedrich‐Alexander‐Universität Erlangen‐Nürnberg, Erlangen, Germany).

### Molecular docking approach

Putative t‐DCA binding sites in the transmembrane region of ASIC1 were predicted using molecular docking software AutoDock Vina (Trott and Olson 2010). The structure of the chicken ASIC1 in its open conformation (PDB: 4NTX; [Baconguis et al. 2014]) was modified by including the polar hydrogen atoms. The grid box defining the docking boundaries was assigned using AutoDockTools 1.5.6 (Sanner 1999; Morris et al. 2009). Structure of t‐DCA was extracted from ZINC version 12 database (ZINC ID: ZINC04282168; [Irwin and Shoichet 2005]). The calculation of the total energy of interaction (MolDock Score [Thomsen and Christensen 2006] between ASIC1 and t‐DCA was made using Molegro Molecular Viewer 2.5.

### Statistical methods

Statistical significance was assessed by the one‐way repeated measures ANOVA with Bonferroni post hoc test or Student's *t*‐test using Graph Pad Prism 5.04. Data of concentration‐dependent stimulation of proton‐activated ASIC1a currents by t‐DCA, pH‐dependent activation and steady‐state desensitization of ASIC1a were normalized as indicated in corresponding figures and fitted using the following equation:

(1) $$Q_{\mathrm{norm}}=Q_{\mathrm{norm},\min}+\left(Q_{\mathrm{norm},\max}-Q_{\mathrm{norm},\min}\right)/\;\left(1+{\left(C_{50}/\;C\right)}^{\mathrm{Hill}}\right),$$

where $Q_{\mathrm{norm},\min}$ is the minimal normalized current integral, $Q_{\mathrm{norm},\max}$ is the maximal normalized current integral, C is t‐DCA concentration or pH, $C_{50}$ is the t‐DCA concentration or pH at which half‐maximal response occurs, and Hill is the Hill coefficient. *N* indicates the number of different batches of oocytes, and *n* indicates the number of individual oocytes studied.

## Results and Discussion

**Figure 1:**
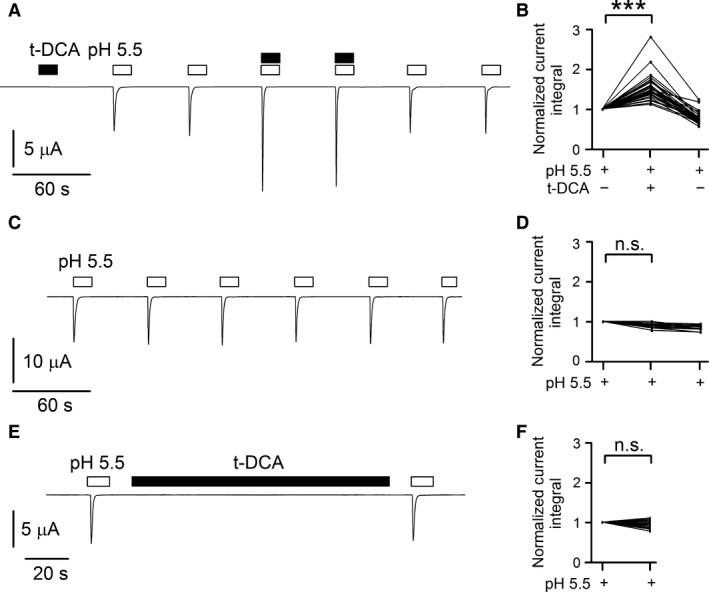
ASIC1a‐mediated whole‐cell currents elicited by pH 5.5 were significantly increased in the presence of t‐DCA but were unaffected by t‐DCA pretreatment. (A, C, E), Representative whole‐cell current traces recorded in human ASIC1a expressing oocytes. Bath pH was 7.4 except for time intervals indicated by open bars during which bath pH was switched to 5.5. t‐DCA (500 μmol/L) was present in the bath solution as indicated by black bars. In the control experiment shown in *C* the 3rd and 4th pH 5.5 responses were elicited using a solution from a different reservoir to confirm the reproducibility of the responses. (B, D, F), Summary of results from similar experiments as shown in A (*n *=* *34; *N* = 6), *C* (*n *=* *15; *N* = 3), and E (*n *=* *25; *N* = 2), respectively. Lines connect data points obtained in the same experiment. To quantify current responses, the integral of the inward current elicited by pH 5.5 below baseline was determined for each response. For the summary data shown in B and D mean current integral values of the 3rd and 4th response and of the 5th and 6th response were normalized to the mean current integral of the first two responses (Normalized current integral). For the summary data shown in F the current integral value of the 2nd response (after t‐DCA) was normalized to the current integral of the 1st response (before t‐DCA). *** p < 0.001; n.s. not significant; one‐way repeated measures ANOVA with Bonferroni post hoc test was used in B and D; Student's *t*‐test was used in F.

First, we tested whether common human bile acids can elicit ASIC1a‐mediated currents at pH 7.4 in oocytes expressing human ASIC1a. We have recently shown that under similar conditions 250 μmol/L t‐DCA were sufficient to cause a substantial stimulation of ENaC currents in oocytes expressing human *αβγ*‐ or *δβγ*ENaC (Ilyaskin et al. 2016). In the representative experiment shown in Figure 1A, t‐DCA was applied in a concentration of 500 μmol/L while maintaining bath pH at 7.4 for the first application of t‐DCA at the beginning of the experiment. Application of t‐DCA at pH 7.4 did not result in a current response which indicates that t‐DCA *per se* does not activate ASIC1a. This is consistent with a previous report showing that ursodeoxycholic acid (2 mmol/L) failed to activate rat ASIC1a, whereas it potently activated rat BASIC (Schmidt et al. 2014). Lowering extracellular pH is an established maneuver to activate ASIC1a which starts to open at pH ~6.9 and to saturate at pH ~6.0 with half maximal activation occurring at a pH ~6.5 (Gründer and Chen 2010; Gründer and Pusch 2015; Kellenberger and Schild 2015). Indeed, switching bath pH from 7.4 to 5.5 resulted in a typical transient inward current response confirming functional expression of ASIC1a (Fig. 1A). The transient nature of the current response induced by pH 5.5 is due to channel desensitization. Return to pH 7.4 allowed recovery from desensitization. Thus, a second exposure to pH 5.5 elicited a current response similar in size and shape to that observed with the first application of pH 5.5. This finding is consistent with previous reports that in the oocyte expression system transient ASIC1a currents of reproducible magnitude can be elicited repeatedly by low pH, provided channels are allowed to recover from desensitization (Pfister et al. 2006; Joeres et al. 2016). We hypothesized that bile acids may not activate ASIC1a *per se* but may modify channel activation by protons. Therefore, we tested the effect of t‐DCA on ASIC1a currents activated by low pH. Interestingly, current responses elicited by pH 5.5 were increased in the presence of t‐DCA (Fig. 1A, 3rd and 4th current response to pH 5.5). This indicates that t‐DCA potentiates the activation of ASIC1a by low pH. The effect of t‐DCA on ASIC1a was fully reversible (Fig. 1A, 5th and 6th current response to pH 5.5). Summarized results from similar experiments indicate that on average t‐DCA increased the ASIC1a current response to pH 5.5 by a factor of 1.55 ± 0.05 (p < 0.001, *n *=* *34; *N* = 6) (Fig. 1B). In similar experiments we also tested the effects of 500 μmol/L t‐CA or t‐CDCA. Like t‐DCA, application of t‐CA or t‐CDCA had no effect on baseline currents in ASIC1a expressing oocytes but significantly increased the average ASIC1a current responses to pH 5.5 by a factor of 1.15 ± 0.02 (*P* < 0.001, *n *=* *20; *N* = 3) or 1.97 ± 0.08 (*P* < 0.001, *n *=* *20; *N* = 3), respectively. Importantly, ASIC1a‐mediated current responses to repeated pH 5.5 pulses remained stable (Fig. 1C, D) in time‐matched control experiments. Bile salts may affect ASIC1a function by altering the properties of the lipid bilayer. We tested whether a prolonged (2 min) exposure to t‐DCA at pH 7.4 increased the subsequent current response to a pulse of pH 5.5 applied in the absence of t‐DCA. As illustrated by the representative current recording in Figure 1E and as summarized in Figure 1F, ASIC1a current responses before and after prolonged t‐DCA exposure were not significantly different. The finding that an effect of t‐DCA on ASIC1a is only observed when a pulse of pH 5.5 is applied in the presence of t‐DCA, favors the hypothesis that the t‐DCA effect is mediated primarily by an interaction with the channel itself and not by an effect of t‐DCA on the membrane. However, we cannot rule out the possibility that t‐DCA acutely interacts also with the lipid environment of the channel.

**Figure 2:**
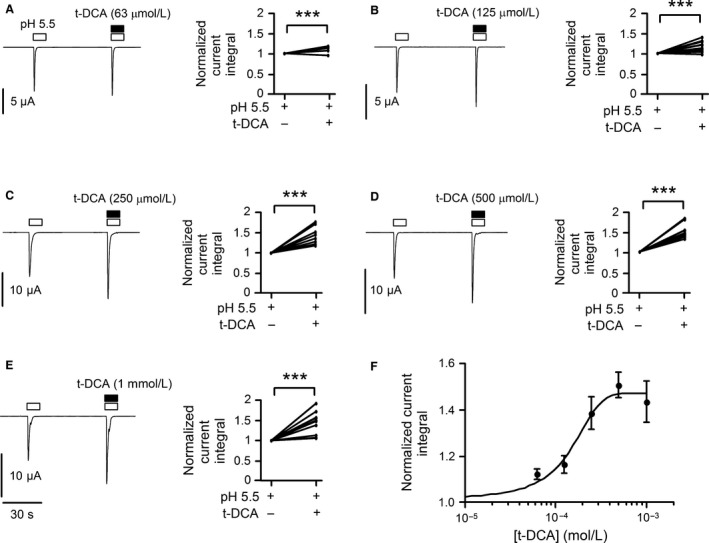
Concentration‐dependent potentiation of proton‐activated ASIC1a currents by t‐DCA. (A–E), Representative whole‐cell current traces recorded in human ASIC1a expressing oocytes exposed to pulses of pH 5.5 in the absence and presence of t‐DCA in concentrations as indicated; data from similar experiments are summarized to the right of each trace. Bath pH was 7.4 except for the time intervals indicated by open bars during which bath pH was switched to 5.5. Lines connect data points obtained in the same experiment. The current integral value of the 2nd response (with t‐DCA) was normalized to the current integral of the 1st response (without t‐DCA). (F), Concentration–response relationship of the stimulatory effect of t‐DCA on proton‐activated ASIC1a currents. Data (mean ± SE) from the same experiments as shown in A–E were fitted to equation** (1) (*n *=* *10; *N* = 2). ***P < 0.001; Student's *t*‐test.

To estimate the apparent affinity of ASIC1a to t‐DCA, we tested the potentiating effect of t‐DCA using five different concentrations (63 μmol/L; 125 μmol/L; 250 μmol/L; 500 μmol/L; 1 mmol/L) (Fig. 2). The summary of the data demonstrated that a saturating concentration of t‐DCA was reached at 500 μmol/L and the estimated EC_50_ averaged 156 ± 22 μmol/L (*n *=* *10; *N* = 2).

**Figure 3:**
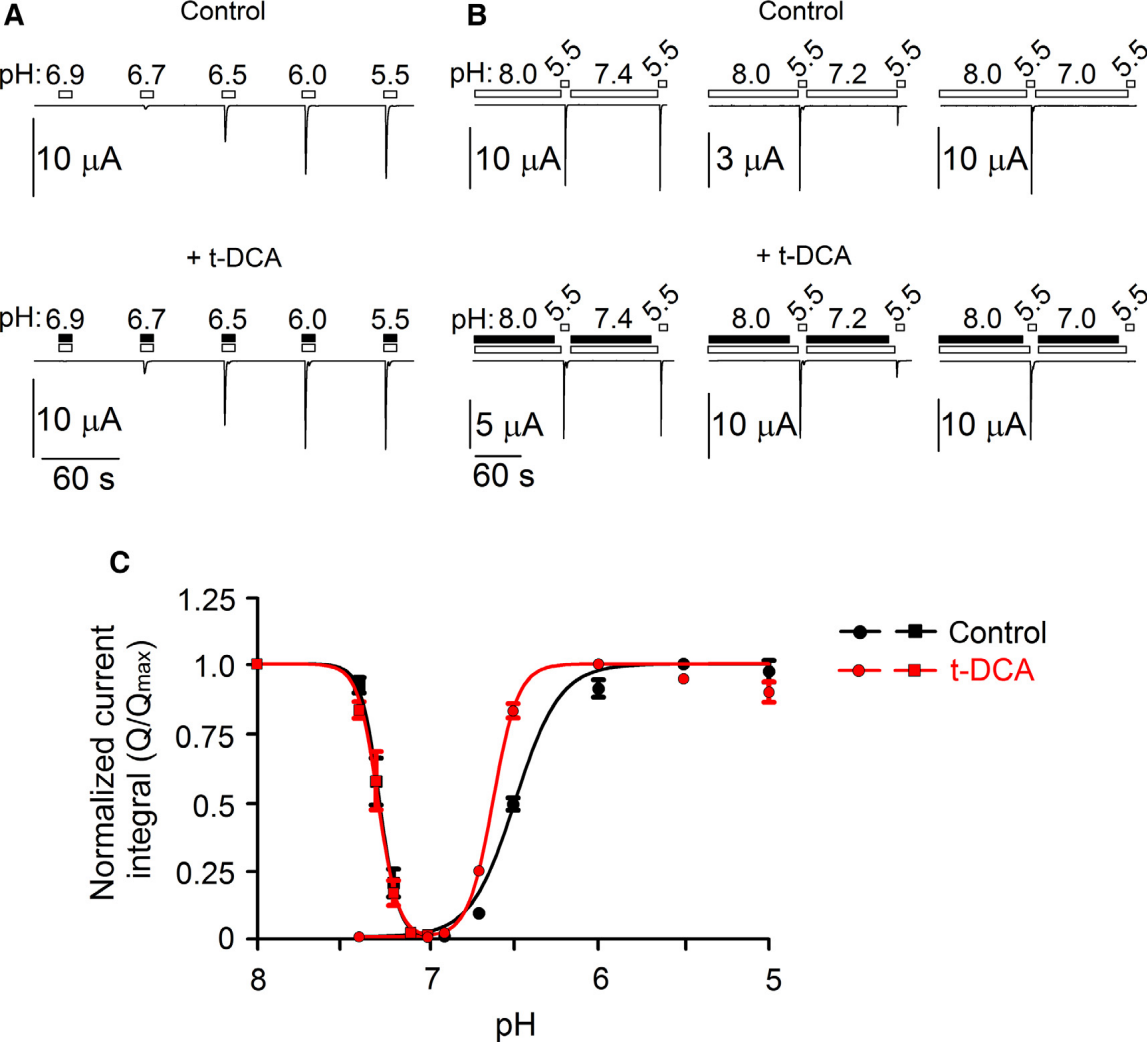
pH dependence of ASIC1a activation and steady‐state desensitization under control conditions and in the presence of t‐DCA. (A), Representative whole‐cell current traces recorded in human ASIC1a expressing oocytes demonstrating the pH‐dependent channel activation. Changes of bath pH from 7.4 to different values ranging from 6.9 to 5.5 are indicated by open bars and were performed under control conditions (*upper panel*) or in the presence of 500 μmol/L t‐DCA as indicated by black bars (*lower panel*). (B), Representative whole‐cell current traces recorded in human ASIC1a expressing oocytes demonstrating the pH‐dependent steady‐state desensitization of the channel under control conditions (*upper panel*) and in the presence of 500 μmol/L t‐DCA as indicated by black bars (*lower panel*). Changes of bath pH are indicated by open bars. After a 2 min exposure to pH 8.0 a first pulse of pH 5.5 was applied followed by a 2 min exposure to pH 7.4, 7.2, or 7.0 and a second pulse of pH 5.5 as indicated. (C), pH‐dependent ASIC1a activation curve under control conditions (●) or in the presence of t‐DCA (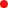) and pH‐dependent ASIC1a steady‐state desensitization curve under control conditions (■) or in the presence of t‐DCA (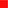). For activation curves the current integral values (*Q*) elicited by pulses of pH were normalized to the maximal value (*Q*_max_) observed in the corresponding recording. *Q*_max_ was observed at pH 5.5 or at pH 6.0 in the absence or presence of t‐DCA, respectively. For the steady‐state desensitization curve the current integral value of the 2nd pulse of pH 5.5 (*Q*, after incubation at different pH values ranging from 7.4 to 7.0) was normalized to the current integral value of the 1st pulse of pH 5.5 (*Q*_max_, after incubation at pH 8.0). Data (mean ± SE) were fitted using equation (1) (6 ≤ *n *≤* *20, *N* = 3 for activation curve; 10 ≤ *n ≤ *12; *N* = 3 for desensitization curve).

To examine whether t‐DCA affects the affinity of ASIC1a to protons, we investigated the pH dependence of ASIC1a activation and steady‐state desensitization under control conditions and in the presence of t‐DCA (Fig. 3). Importantly, the apparent proton affinity of ASIC1a increased in the presence of t‐DCA as indicated by a significant left shift of the pH‐activation curve (Fig. 3A, C). pH_0.5_ of ASIC1a activation was significantly increased from 6.49 ± 0.02 under control conditions to 6.62 ± 0.02 in the presence of t‐DCA (*P* < 0.001; 6 ≤ *n ≤ *20; *N* = 3). Moreover, the Hill coefficient of the activation curve was significantly higher in the presence (5.92 ± 0.89) than in the absence (3.32 ± 0.76) of t‐DCA (*P* < 0.05; 6 ≤ *n ≤ *20; *N* = 3). This suggests that t‐DCA may increase the cooperativity of proton binding to ASIC1a. Interestingly, t‐DCA had no significant effect on the pH dependence of ASIC1a steady‐state desensitization (Fig. 3B, C). Neither pH_0.5_ of ASIC1a steady‐state desensitization nor the Hill coefficient were affected by t‐DCA. pH_0.5_ of ASIC1a steady‐state desensitization averaged 7.28 ± 0.01 and 7.29 ± 0.01 in the absence and presence of t‐DCA, respectively. The corresponding Hill coefficients were 8.09 ± 0.44 and 7.39 ± 0.59 (n.s.; 10 ≤ *n ≤ *12; *N* = 3). We conclude that an increased proton affinity of ASIC1a may contribute to the observed potentiating effect of bile acids on proton‐activated ASIC1a currents. However, the potentiating effect of t‐DCA persists at pH 5.5 where the channel is maximally activated by protons (see Fig. 1). This suggests that additional mechanisms are involved. The finding that t‐DCA does not affect the pH dependence of ASIC1a steady‐state desensitization indicates that bile acids modulate ASIC1a in the open state of the channel.

**Figure 4:**
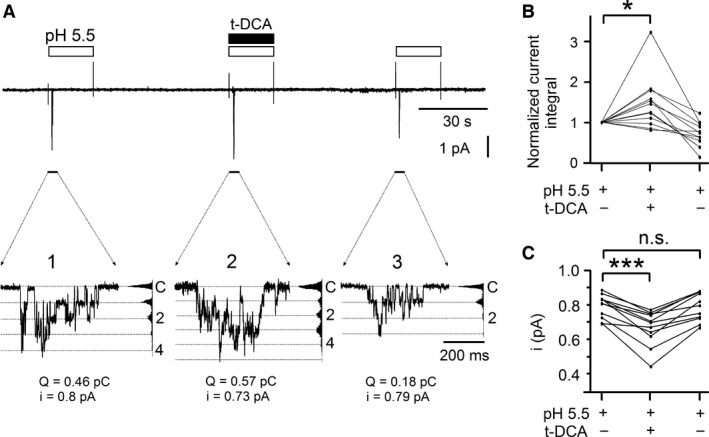
t‐DCA increased proton‐activated ASIC1a single‐channel activity in outside‐out patches and slightly reduced single‐channel current amplitude. (A), Representative single‐channel current recording obtained at a holding potential of −70 mV from an outside‐out patch of an ASIC1a expressing oocyte. Bath pH was 7.4 except for three time intervals indicated by open bars during which bath pH was switched to 5.5. t‐DCA (500 μmol/L) was present in the bath solution as indicated by the black bar. The current level at which all channels are closed (C) was determined at pH 7.4. The *insets* (1, 2, and 3) show the indicated segments of the continuous current trace on an expanded time scale. Binned current amplitude histograms are shown on the right side of the *insets* and were obtained from the corresponding parts of the trace to calculate single‐channel current amplitude (*i*). Dotted lines in the *insets* indicate channel open levels. To quantify the responses, the current integral (*Q*) of the single‐channel currents activated by pH 5.5 was determined for each response. (B, C), Summary of results from similar experiments as shown in *A*. Lines connect data points obtained in the same experiment. In *B* (*n *=* *12; *N* = 4) values were normalized to the initial response to pH 5.5 (Normalized current integral). In *C* the effect of t‐DCA on single‐channel current amplitude is summarized (*n *=* *11; *N* = 4).*P < 0.05; ***P < 0.001; n.s., not significant; one‐way repeated measures ANOVA with Bonferroni post hoc test.

To investigate the bile acid effect on ASIC1a at the single‐channel level, we performed outside‐out patch‐clamp recordings. Figure 4A shows a representative recording from an outside‐out patch excised from an oocyte expressing ASIC1a. Initial exposure of the patch to pH 5.5 elicited transient single‐channel activity typical for ASIC1a (Zhang and Canessa 2002; Yang and Palmer 2014) with up to four apparent channel levels (Fig. 4A, inset 1). The integral of the current activated by this initial exposure to pH 5.5 equaled 0.46 pC. Subsequently, the patch was re‐exposed to pH 5.5 in the presence of t‐DCA. This also elicited ASIC1a channel activity, but the number of apparent channels in the patch was increased to five, and the integral of the activated current was increased to 0.57 pC (Fig. 4A, inset 2). In contrast, a third exposure to pH 5.5 performed in the absence of t‐DCA (Fig. 4A, inset 3) elicited a response similar to the initial response to pH 5.5. On average, t‐DCA significantly increased the pH 5.5 induced ASIC1a single‐channel current activity in outside‐out patches by a factor of 1.46 ± 0.19 (*P* < 0.05, *n *=* *12; *N* = 4) (Fig. 4B). This is in good agreement with the results obtained in our whole‐cell recordings. Interestingly, in the recording shown in Figure 4A (insets 1–3) the stimulatory effect of t‐DCA on proton activated ASIC1a single‐channel activity was accompanied by a small and reversible reduction in the single‐channel current amplitude (i) from 0.8 pA to 0.73 pA. On average, t‐DCA significantly reduced ASIC1a single‐channel current amplitude from 0.79 ± 0.02 pA to 0.67 ± 0.03 pA (*P* < 0.001, *n *=* *11; *N* = 4) (Fig. 4C). This suggests that bile acids may modulate ASIC1a channel function by interacting with a site close to the channel pore. The precise mechanism by which t‐DCA reduces single‐channel current amplitude remains to be elucidated and may involve an increase in channel flickering.

**Figure 5:**
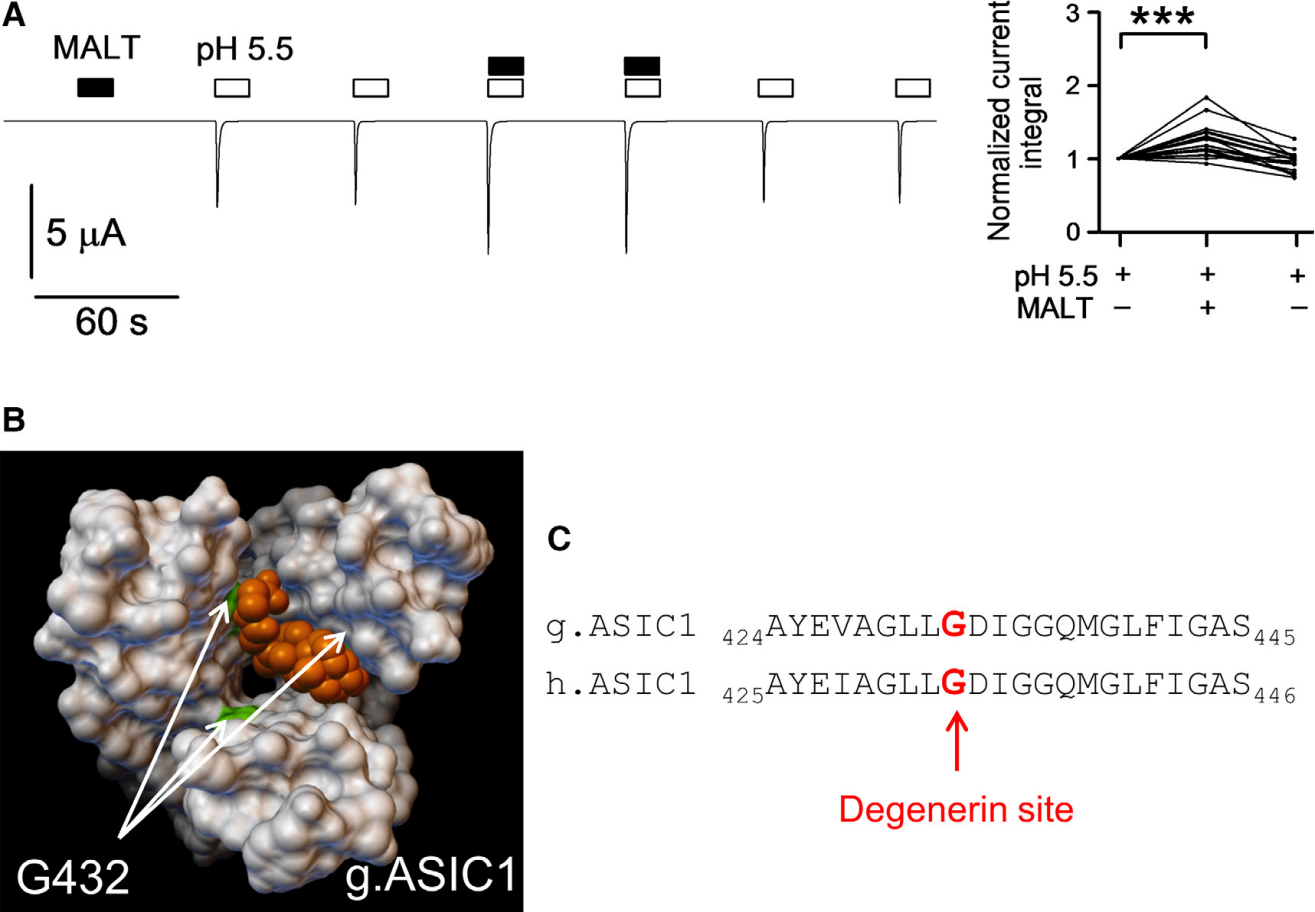
Molecular docking approach using the open state crystal structure of ASIC1 predicted binding of t‐DCA to the channel pore. (A), Representative whole‐cell current trace recorded in an oocyte expressing human ASIC1a. The oocyte was exposed to pH 5.5 and n‐dodecyl‐*β*‐D‐maltoside (MALT, 10 μmol/L) as indicated by open and black bars, respectively. Data from similar experiments are summarized to the right of the trace like in Figure 1B and D as normalized current integrals (*n *=* *20; *N* = 3). (B), Molecular surface representation of the transmembrane domains of chicken ASIC1 (Baconguis et al. 2014) viewed from the extracellular side with a t‐DCA molecule (orange color) positioned at the site predicted by molecular docking approach. In the docking model shown the putative interaction site of t‐DCA corresponds to the site where maltoside was cocrystallized with ASIC1 (Jasti et al. 2007). The arrows indicate the position of glycine residue 432 (G432; highlighted in green) in each ASIC1a subunit. G432 is the amino acid residue with the highest contribution to the total energy of interaction between ASIC1 and t‐DCA. (C), Sequence alignment of chicken (g.ASIC1) and human (h.ASIC1a) corresponding to the first part of the TM2. The homologous amino acid residues G432 in g.ASIC1 and G433 in h.ASIC1a are indicated by bold characters highlighted in red.

**Figure 6:**
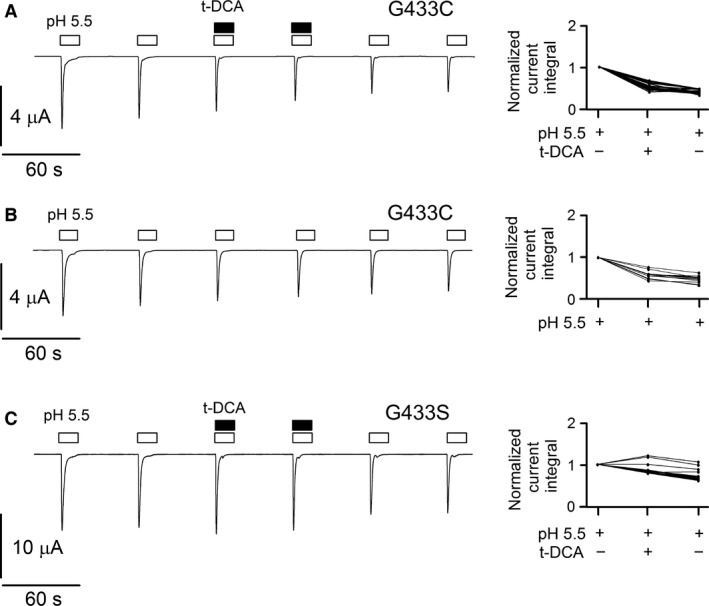
Substituting the glycine residue G433 by cysteine or serine abolished or reduced the stimulatory effect of t‐DCA on proton‐activated ASIC1a currents, respectively. (A, B, C), *Left panels:* Representative whole‐cell current traces recorded in oocytes expressing the G433C (A and B) or G433S (C) mutant ASIC1a. Experiments were performed and analyzed as described for the experiments shown in Figure 1A–D. *Right panels:* Summary of data from similar experiments as shown in the left panel: A (*n *=* *22; *N* = 3), B (*n *=* *13; *N* = 3), C (*n *=* *22; *N* = 3).

High‐resolution crystal structure information is available for chicken ASIC1a (Jasti et al. 2007; Gonzales et al. 2009; Baconguis and Gouaux 2012; Dawson et al. 2012; Baconguis et al. 2014). Interestingly, n‐dodecyl‐*β*‐D‐maltoside molecules were cocrystallized within the pore region of ASIC1 (PDB: 2QTS; [Jasti et al. 2007; Ilyaskin et al. 2016]). Bile acids are known to have amphiphilic properties and can behave as detergents (Helenius and Simons 1975). Therefore, we hypothesized that maltoside may modulate ASIC1a activation by low pH in a similar manner as bile acids. Indeed, as illustrated in Figure 5A, exposing an ASIC1a expressing oocyte to n‐dodecyl‐*β*‐D‐maltoside (10 μmol/L) at pH 7.4 did not cause any current response *per se,* but maltoside significantly increased proton‐activated ASIC1a currents mimicking the effect of t‐DCA. On average, the ASIC1a‐mediated current response elicited by pH 5.5 was increased in the presence of maltoside by a factor of 1.23 ± 0.05 (*P* < 0.001; *n *=* *20; *N* = 3) (Fig. 5A). This finding suggests that maltoside and bile acids may interact with the pore region of ASIC1 at a similar site. To identify a putative bile acid binding site in the pore region of ASIC1, a molecular docking approach was used (Trott and Olson 2010). We hypothesized that bile acids interact with the open state of the channel, because our functional data demonstrated that bile acids did not activate ASIC1a *per se* but increased proton‐activated ASIC1a currents. Thus, for the molecular docking approach we used the crystal structure of chicken ASIC1 in its open conformation (PDB: 4NTX; [Baconguis et al. 2014]). Computer simulations predicted that t‐DCA binds to the pore region of ASIC1 (Fig. 5B) at the site where maltoside was cocrystallized. Estimating the energy of interaction (MolDock Score) between the t‐DCA molecule and the channel revealed that the glycine residue in position 432 (G432) may play a crucial role in t‐DCA binding (Fig. 5B). Protein sequence alignment demonstrated that the second transmembrane domains in chicken and human ASIC1a are highly conserved and that G432 in chicken ASIC1 corresponds to G433 in human ASIC1a (Fig. 5C). Thus, G433 may be a functionally important site for the interaction of bile acids with human ASIC1a. Therefore, we mutated glycine 433 to cysteine (G433C) or serine (G433S) and tested whether these substitutions alter the effect of t‐DCA on ASIC1a. Cysteine or serine were chosen because these substitutes do not change substantially the size and charge of the amino acid side chain. Moreover, in previous studies (Tolino et al. 2011; van Bemmelen et al. 2015) an analogous glycine to cysteine mutation resulted in expression of functional channels with a pH sensitivity similar to that of wild‐type channels. The G433C mutation abolished the stimulatory effect of t‐DCA on the ASIC1a‐mediated current response to pH 5.5 (Fig. 6A). The gradual decline of the magnitude of the mutant ASIC1a currents with repeated exposures to pH 5.5 was also observed in matched control experiments without t‐DCA (Fig. 6B). This latter finding is in good agreement with a previous study which reported enhanced tachyphylaxis of ASIC1a carrying the G433C mutation (Carattino and Della Vecchia 2012). The stimulatory effect of t‐DCA on proton‐activated ASIC1a currents was also reduced by the G443S mutation (Fig. 6C). Our findings that the G433C mutation abolishes and that the G443S mutation reduces the stimulatory effect of t‐DCA suggest that the G433 residue plays an important role in mediating the effect of bile acids to increase ASIC1a activation by low pH. This does not preclude the existence of additional t‐DCA‐binding sites.

The identified putative bile acid binding site in ASIC1a is localized to the extracellular vestibule of the channel in the region called “desensitization gate” (Gonzales et al. 2009; Li et al. 2011). The position G433 corresponds to the “degenerin” site known to be important for the gating of channels belonging to the ENaC/DEG family (Kellenberger and Schild 2015). Recently, we have shown that the degenerin site is also critical for the functional interaction of bile acids with human ENaC (Ilyaskin et al. 2016). Thus, a similar molecular mechanism may be responsible for the modulatory effect of bile acids on ASIC1a function. Comparison of ASIC1 crystal structures in desensitized and open states revealed that opening of the channel by protons widens the extracellular vestibule (Baconguis and Gouaux 2012; Baconguis et al. 2014). Therefore, upon proton‐mediated channel activation the increased size of the vestibule may permit an interaction of bile acids with G433. This may explain the increased ASIC1a‐mediated current response to low pH observed in the presence of bile acids.

Several endogenous and exogenous substances have been described to modify ASIC function (Wemmie et al. 2013). It is conceivable that bile acids may play a role in ASIC regulation under physiological and pathophysiological conditions. Recent evidence suggest that increased ASIC activity in sensory neurons of gastrointestinal tract may contribute to hyperalgesia and colonic hypersensitivity observed in patients with IBS (Irritable bowel syndrome) (Holzer 2015). At present the molecular mechanism of ASIC hyperactivity in IBS remains unclear. It is tempting to speculate that increased bile acid concentration in the gut of IBS patients due to bile acid malabsorption and increased biosynthesis (Hofmann 1999; Wong et al. 2012) may lead to ASIC overstimulation and hence play a role in the pathogenesis of IBS. Moreover, our finding that bile acids and maltoside detergent have similar effects on ASIC1a raises the possibility that additional endogenous amphiphilic substances may modulate ASIC activity. Endogenous substances capable of binding to the degenerin region of the channel may act as local modulators of ASIC function in a tissue‐specific manner but remain to be identified.

In conclusion, our results highlight the potential role of the degenerin region as a regulatory site involved in the functional interaction of bile acids and possibly other naturally occurring amphiphilic substances with ASIC1a.

## Conflict of Interests

The authors declare that they have no conflict of interest with the contents of this article.
